# Slippery Liquid-Like Solid Surfaces with Promising Antibiofilm Performance under Both Static and Flow Conditions

**DOI:** 10.1021/acsami.1c14533

**Published:** 2022-01-31

**Authors:** Yufeng Zhu, Glen McHale, Jack Dawson, Steven Armstrong, Gary Wells, Rui Han, Hongzhong Liu, Waldemar Vollmer, Paul Stoodley, Nicholas Jakubovics, Jinju Chen

**Affiliations:** † School of Engineering, Newcastle University, Newcastle Upon Tyne NE1 7RU, U.K.; ‡ School of Engineering, University of Edinburgh, Edinburgh EH9 3FB, U.K.; § School of Mechanical Engineering, Xi’an Jiaotong University, Xi’an 710054, China; ∥ Centre for Bacterial Cell Biology, Biosciences Institute, Newcastle University, Newcastle Upon Tyne NE2 4AX, U.K.; ⊥ Department of Microbial Infection and Immunity and the Department of Orthopaedics, The Ohio State University, Columbus, Ohio 43210, United States; # National Centre for Advanced Tribology at Southampton (nCATS), National Biofilm Innovation Centre (NBIC), Mechanical Engineering, University of Southampton, Southampton S017 1BJ, U.K.; ¶ School of Dental Sciences, Faculty of Medical Sciences, Newcastle University, Newcastle Upon Tyne NE2 4BW, U.K.

**Keywords:** antibiofilm, slippery polymer surfaces, liquid-like surface, biofilm detachment, surface wetting

## Abstract

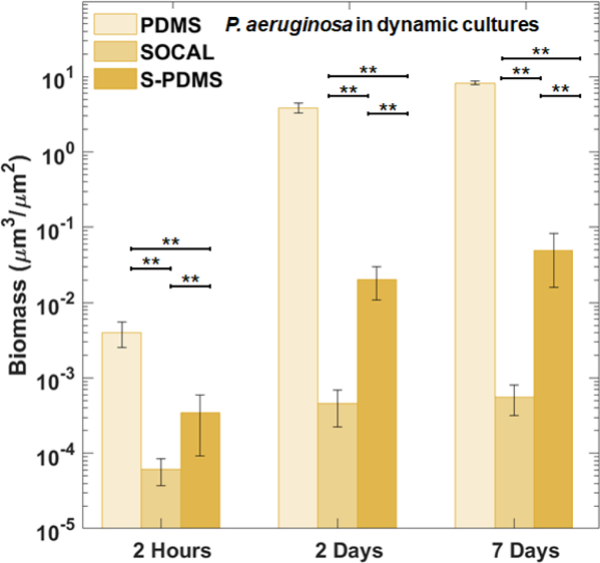

Biofilms are central to some of the most urgent global challenges across diverse fields of application, from medicine to industries to the environment, and exert considerable economic and social impact. A fundamental assumption in anti-biofilms has been that the coating on a substrate surface is solid. The invention of slippery liquid-infused porous surfaces—a continuously wet lubricating coating retained on a solid surface by capillary forces—has led to this being challenged. However, in situations where flow occurs, shear stress may deplete the lubricant and affect the anti-biofilm performance. Here, we report on the use of slippery omniphobic covalently attached liquid (SOCAL) surfaces, which provide a surface coating with short (ca. 4 nm) non-cross-linked polydimethylsiloxane (PDMS) chains retaining liquid–surface properties, as an antibiofilm strategy stable under shear stress from flow. This surface reduced biofilm formation of the key biofilm-forming pathogens *Staphylococcus epidermidis* and *Pseudomonas aeruginosa* by three–four orders of magnitude compared to the widely used medical implant material PDMS after 7 days under static and dynamic culture conditions. Throughout the entire dynamic culture period of *P. aeruginosa*, SOCAL significantly outperformed a typical antibiofilm slippery surface [i.e., swollen PDMS in silicone oil (S-PDMS)]. We have revealed that significant oil loss occurred after 2–7 day flow for S-PDMS, which correlated to increased contact angle hysteresis (CAH), indicating a degradation of the slippery surface properties, and biofilm formation, while SOCAL has stable CAH and sustainable antibiofilm performance after 7 day flow. The significance of this correlation is to provide a useful easy-to-measure physical parameter as an indicator for long-term antibiofilm performance. This biofilm-resistant liquid-like solid surface offers a new antibiofilm strategy for applications in medical devices and other areas where biofilm development is problematic.

## Introduction

Many microorganisms form sessile communities, called biofilms, in self-produced extracellular polymeric substances (EPSs), which often attach to solid surfaces. Biofilm-associated infections have dramatic economic and societal impacts. For instance, it was estimated that biofilm infections cost about $94 billion p.a. in the United States healthcare system.^1^ Moreover, around 6–14% of hospitalized patients suffer from biofilm infections associated with medical devices, such as urinary catheters, peritoneal dialysis catheters, tracheal prostheses, pacemakers, endotracheal tubes, dental implants, and orthopedic implants.^2^ Among these, catheter-associated urinary tract infections (CAUTIs) in hospitals are estimated to cause additional healthcare costs of £1–2.5 billion in the UK alone.^3^ Catheter-related bloodstream infections (CRBSIs) are mainly responsible for nosocomial infection in intensive care units (ICUs), resulting in morbidity, mortality, and significant economic cost.^4,5^

Methods to prevent biofilm formation and growth on medical device surfaces include immobilization of antimicrobial agents^6^ (i.e., antibiotics, peptide, silver particles, or nitric oxide), the use of special surface texture,^7−11^ surface grafting with poly(ethylene glycol) (PEG) or zwitterionic polymers,^12,13^ quaternary ammonium salt-functionalized fluorinated copolymers,^14^ and the use of biofilm-dispersing enzymes.^15^ All anti-biofilm surfaces have their own challenges. For instance, surfaces based on antimicrobial agents lose their efficacy over time, and they can potentially trigger antimicrobial resistance.^7,15^ Antibiofilm surface textures have either nanospears to mechanically rupture the bacterial cell wall, causing cell lysis,^7−9,11^ or they trap air within microstructures or nanostructures to restrict direct contact between the solid surface and microorganisms.^16−18^ For the former, the fast-growing surviving bacteria mask the nanospear structures, which restricts their long-term antimicrobial efficiency.^7^ For the latter, the anti-biofilm efficacy strongly depends on the lifetime of the non-wetting (Cassie) state, which is often short in submerged environments.^16,19,20^ The antibiofilm performance of surfaces grafted with PEG or zwitterionic polymers is also transient because the adsorption of proteins and surfactants secreted by bacteria can mask the underlying surface.^21^ Although these surfaces are promising, new developments are required to improve their durability.

Recently, anti-biofilm approaches have been developed based on endowing the surface with a liquid lubricant. There are many physical and chemical methods which can potentially maintain a stable lubricant layer by capillary forces, chemical interactions, swelling, and employing microcapsules to lock the lubricants.^22^ Typically, a porous or textured solid surface is infused with a liquid lubricant locked into the structure with capillary forces to create a stable hemi-solid/hemi-liquid surface^23^ or a continuous lubricant coating (a slippery liquid-infused porous surface—SLIPS).^24,25^ Another complementary liquid lubricant-based approach uses a polydimethylsiloxane (PDMS) matrix infused with silicone oil (known as S-PDMS), causing it to swell and locking in a large reservoir of oil in the polymer chains.^26,27^ These liquid lubricant-based surfaces inhibit the surface attachment of bacteria and have great promise as antibiofilm surfaces.^27−37^ However, the potential loss of lubricants through repeated usage or shear^38−40^ remains a key limiting factor to wider adoption as a practical solution. In clinical settings, this may be a safety risk for patients.

In the present work, we report an anti-biofilm surface strategy using liquid-like solid surfaces, where the risk of lubricant loss is removed. Our coating is used as a slippery omniphobic covalently attached liquid-like (SOCAL) surface, obtained through acid-catalyzed graft polycondensation of dimethyldimethoxysilane, first proposed by Wang and McCarthy as an ultra-slippery non-pinning surface for sessile droplets.^41,42^ This SOCAL surface displays wetting properties similar to SLIPS through its grafted PDMS coating that behaves as a liquid phase approximately 150 °C above its glass transition temperature.^41,43^ The wetting properties of SOCAL coatings have been increasingly cited, but only a handful of groups have implemented the techniques and successfully fabricated SOCAL with contact angle hysteresis (CAH) below 3°.^41,43^ The optically transparent SOCAL surface has often been discussed in the context of superhydrophobic surfaces with interesting surface wetting properties.^41,43^ No work has been carried out to assess and understand its antibiofilm performance.

We demonstrate antibiofilm performance of SOCAL, as a permanently bound liquid-like solid lubricant surface, in both static cell culture without flow and dynamic culture with continuous flow. The anti-biofilm performance against two major nosocomial pathogens, *Staphylococcus epidermidis* and *Pseudomonas aeruginosa,* is presented. We also discuss possible new mechanisms by which oil depletion of S-PDMS can affect the colonization of *S. epidermidis* and *P. aeruginosa*in a different way.

## Results

### Surface Preparation

**Figure 1:**
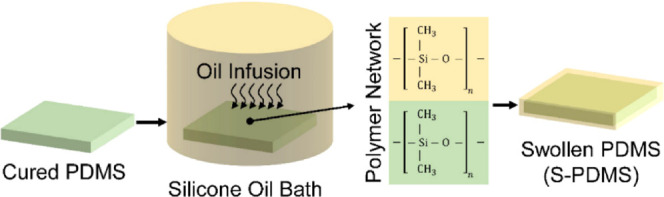
Schematic diagram for preparing S-PDMS prepared by infusing silicone oil into PDMS (silicone).

SOCAL was prepared following the procedure of Wang and McCarthy^41^ as implemented by Armstrong.^43^ This used dip coating of a reactive solution of isopropanol, dimethyldimethoxysilane, and sulfuric acid on plasma-treated glass and drying in a controlled-humidity environment to cause an acid-catalyzed graft polycondensation of dimethyldimethoxysilane, resulting in a liquid-like polymer coating. Figure 1 displays the schematic diagram for preparing S-PDMS. The detailed protocols for sample preparations are provided in the Methods. To ensure that PDMS was a suitable control for SOCAL surfaces, the surface chemistry of the two materials was assessed by X-ray photoelectron spectroscopy (XPS) spectrum analysis (see Figure S1 in Supporting Information). The XPS of the SOCAL coating prepared by such a dip coating approach was similar to that of PDMS.^44^ Our atomic force microscopy (AFM) nanoindentation tests with an empirical model have shown that an estimated modulus of SOCAL is about 8.8 kPa, which should be treated as an upper bound because the substrate from the underlying glass cannot be completely removed.^45^

### Surface Wettability

The static contact angle (CA) and the CAH are important parameters for water repellency and the ability of a surface to shed water, which could be correlated with the repellency to bacterial adhesion. Both CA and CAH of water droplets on PDMS (control sample), SOCAL, and S-PDMS are summarized in Table 1. S-PDMS and SOCAL have a CA of 100.0 ± 1.4° and 104.9 ± 1.6°, respectively, which is consistent with previous measurements^37,43^ and theoretical predictions based on a surface free energy approach.^46,47^ The measured oil thickness of the S-PDMS surface is estimated to be 26.1 ± 5.3 μm. The thickness of SOCAL measured by ellipsometry was (3.9 ± 0.6) nm, which is consistent with that previously reported.^41^ Such a thickness of SOCAL is important for achieving CAH below 3°.^41^ Both SOCAL surfaces and S-PDMS have CAH an order of magnitude less than that of PDMS, which implies an order of magnitude reduction in force to induce droplet shedding by motion along the surface,^48^ thereby confirming its slippery surface properties.

**Table 1:** Static CA and the CAH of Water Droplets on Different Surfaces^a^

surface	CA (°)	advancing angle (°)	receding angle (°)	contact angle hysteresis (°)
PDMS	117.5 ± 1.1	116.8 ± 1.5	95.4 ± 1.3	21.4 ± 2.1
SOCAL	104.9 ± 1.6	105.1 ± 0.8	103.0 ± 1.3	2.0 ± 1.0
S-PDMS	100.3 ± 1.4	99.1 ± 3.2	95.9 ± 2.4	3.2 ± 0.7

a Data represent the mean and SD of five independent measurements.

**Figure 2:**
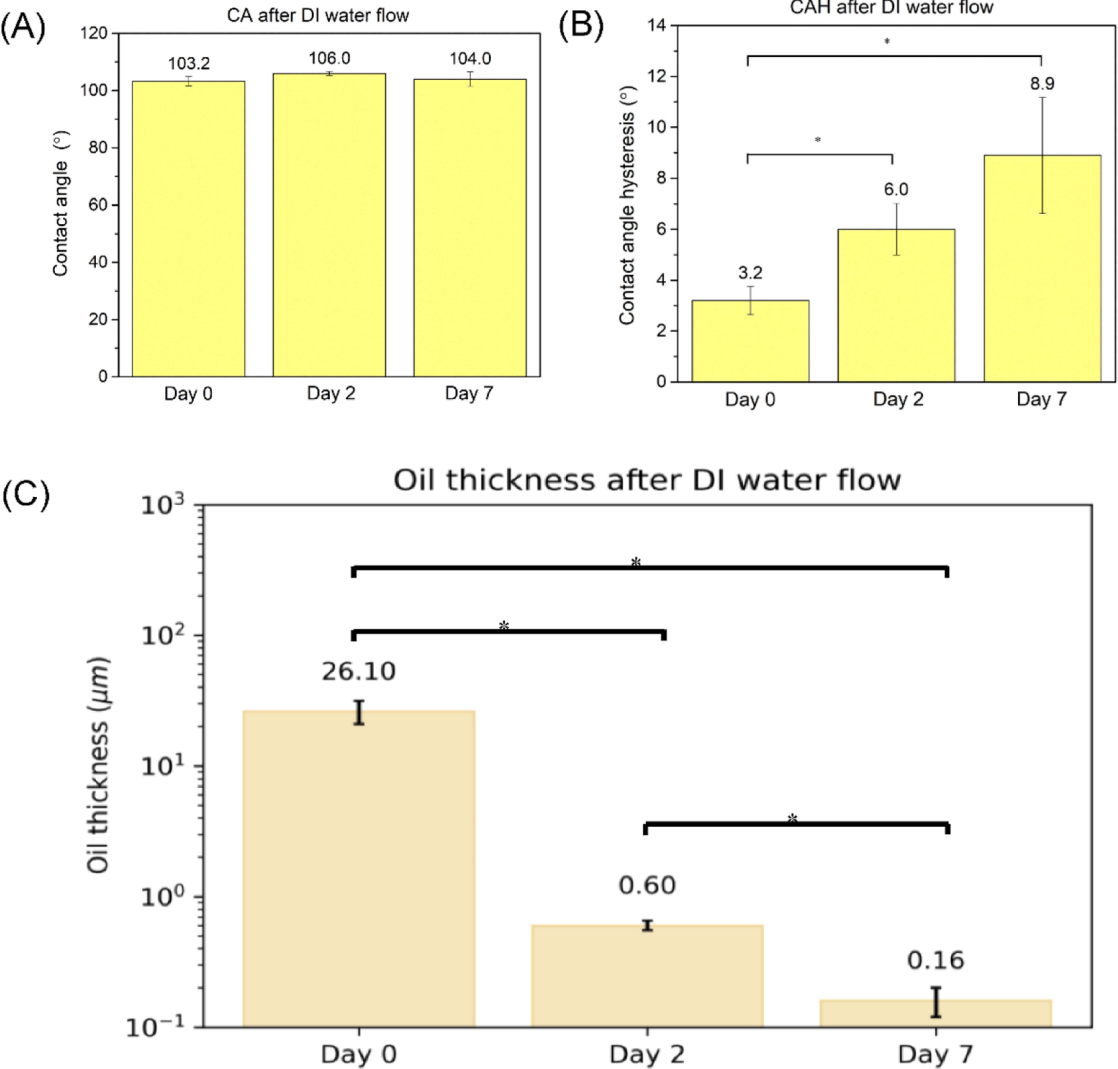
(A) Oil thickness atop S-PDMS and the corresponding (B) CA and (C) CAH subjected to the continuous flow (τ_w_ = 0.007 Pa) for 2 days and 7 days.*p < 0.05.

As S-PDMS was reported to suffer from oil loss in continuous flow,^40^ we also measured the oil loss and investigated how the oil loss may affect the CA and CAH. The key results are presented in Figure 2. After continuous flow (τ_w_ = 0.007 Pa) for 7 days, for S-PDMS, CA remained unchanged but CAH increased significantly to an average of 8.9°, which is associated with oil loss (see Figure 2). In contrast, there was no detectable change of CA and CAH for SOCAL surfaces.

### Anti-biofilm Tests against *S. epidermidis*

**Figure 3:**
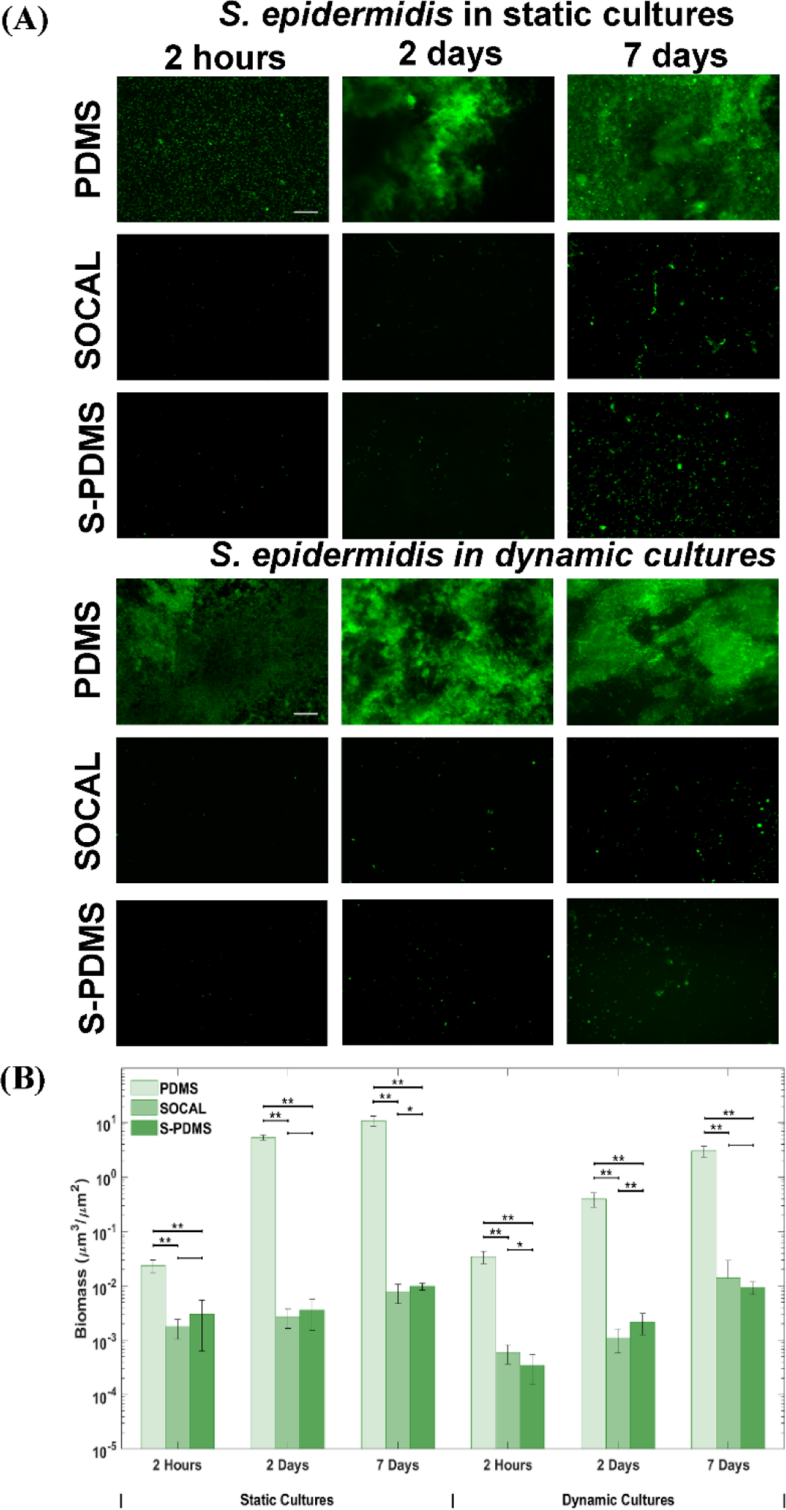
(A) Representative fluorescent images and (B) biomass of the growth of *S. epidermidis* FH8 on PDMS, SOCAL, and S-PDMS for 2 h, 2 days, and 7 days in static cell culture and dynamic cell culture. Scale bar = 50 μm for all images. In all cases, 15 images were analyzed for each surface from three independent experiments. Values presented are mean ± SD. *p < 0.05 and **p < 0.001.

We started by examining the growth of *S. epidermidis* FH8, a recent clinical isolate from a mucosal biofilm, on PDMS and SOCAL after different culture periods under static conditions. PDMS was used as a comparative control surface because it has surface chemistry characteristics similar to SOCAL. The former is cross-linked PDMS, and the latter is a liquid-like uncross-linked PDMS thin film. To assess the anti-biofilm performance of SOCAL, we also performed the tests on swollen PDMS for comparison. We created a SLIP-type surface using S-PDMS. This gives a large reservoir of oil compared to liquid-infused porous structures (LIPs) and has demonstrated excellent antibiofilm performance in static culture in recent studies.^37^ S-PDMS also has a chemistry similar to PDMS and SOCAL, so any impact from surface chemistry will likely be very similar between each of the surfaces. Figure 3A displays the fluorescence images after growth of *S. epidermidis* for 2 h, 2 days, and 7 days on the different surfaces. After 2 h, the control PDMS surface was covered with bacteria with some bacterial aggregates or clusters. However, only sparse and isolated bacterial cells were present on SOCAL and S-PDMS. After 2 days of culture, a large amount of biofilm had formed on PDMS; however, there was only sparse coverage of single cells on SOCAL and S-PDMS. After 7 days, a thick biofilm had formed on PDMS, while only limited bacterial clusters were observed on SOCAL and S-PDMS (Figure 3A). By quantifying the biomass on these surfaces based on fluorescence imaging, it was found that SOCAL and S-PDMS significantly reduced initial bacterial attachment (2 h) by 92 ± 3% and 87 ± 3% (Figure 1B), respectively. After 2 days, both SOCAL and S-PDMS resulted in three orders of magnitude biomass reduction compared to PDMS (*p* = 1.3 × 10^–15^), while after 7 days, the total biomass of the SOCAL and S-PDMS was three orders of magnitudes less than that of PDMS (*p* = 3 × 10^–11^) (Figure 3B).

For the dynamic bacterial culture with continuous flow, flow conditions resulting in a wall shear stress (τ_w_) of 0.007 Pa were chosen to match the flow conditions present in urinary catheters.^49^*S. epidermidis* biofilms grew substantially with time on PDMS (Figure 3A). However, throughout the experiment (up to 7 days), only sparse and isolated bacteria (with no visible EPSs) were observed on the SOCAL and S-PDMS surfaces under identical flow conditions. Compared to PDMS control samples, after 2 h, the SOCAL and S-PDMS surfaces resulted in 98 ± 1% and 99 ± 1% reduction of bacterial attachment. After 2 days, SOCAL and S-PDMS led to over 360- and 180-fold reductions in biofilm volume compared to PDMS (*p* = 3.2 × 10^–9^), respectively. After 7 days, SOCAL and S-PDMS led to over 200- and 300-fold biofilm volume reductions compared to PDMS (*p* = 9.6 × 10^–11^), respectively (Figure 3B). For the 7 day dynamic culture, there was no significant difference (*p* = 0.26) between biomass on SOCAL and S-PDMS surfaces.

When comparing *S. epidermidis* colonization under static and flow conditions, there was a significant difference for PDMS throughout the 7 day culture period (*p* < 0.001). By contrast, differences between SOCAL and S-PDMS were only significant for the first 2 days. There was no significant difference in *S. epidermidis* colonization after 7 days of colonization under static or flow conditions for either SOCAL (*p* = 0.14) or S-PDMS (*p* = 0.70).

**Figure 4:**
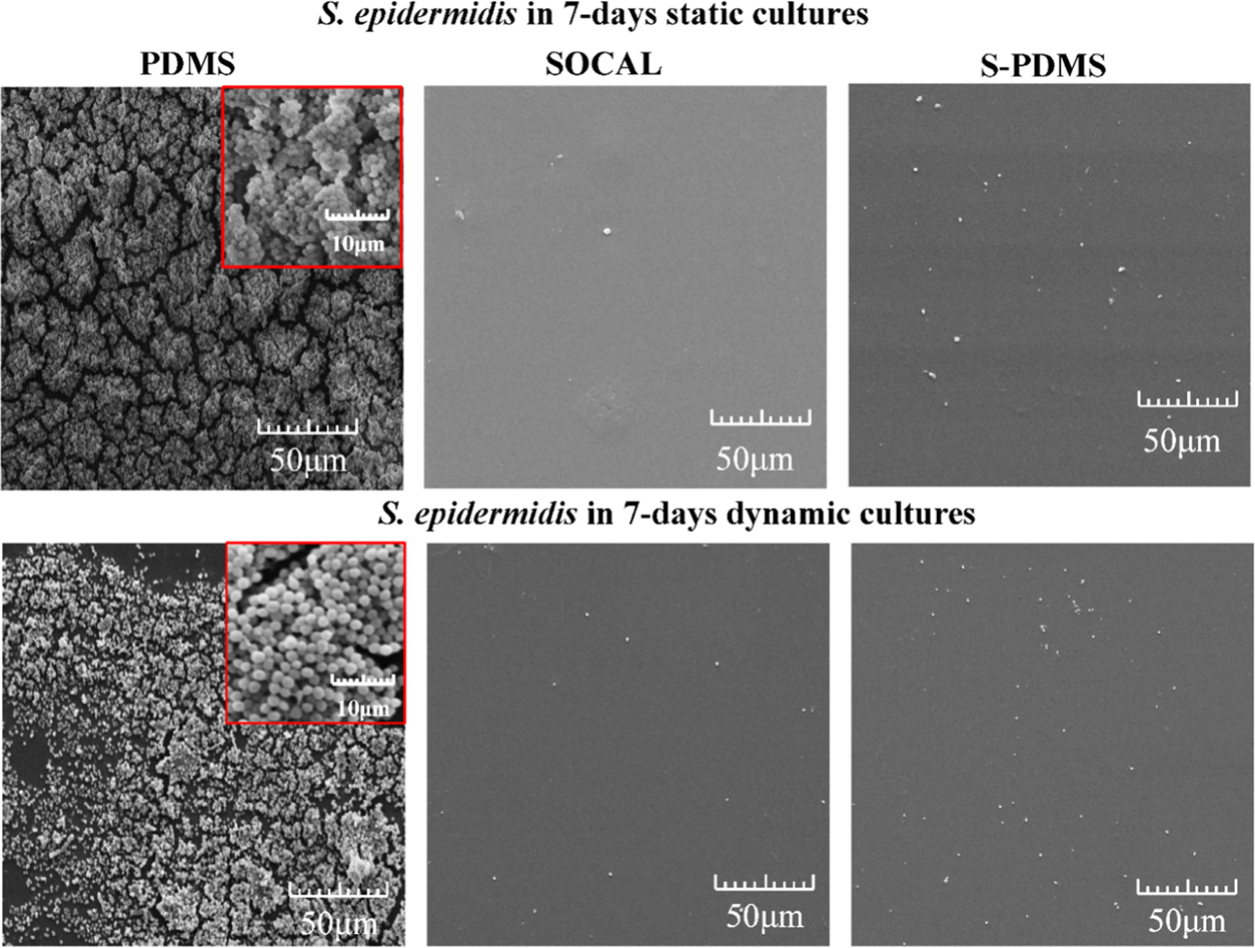
Representative SEM images of 7 day growth of *S. epidermidis* FH8 on PDMS, SOCAL, and S-PDMS in static and dynamic cultures. Dense EPS and biofilm growth were found on PDMS. In contrast, no EPS was found on SOCAL or S-PDMS, and bacteria were also very sparse.

Our scanning electron microscopy (SEM) images (see Figure 4) also confirmed that very dense *S. epidermidis* biofilm growth occurred on PDMS for both 7 day static and dynamic cultures. Only sparse bacteria were found on SOCAL or S-PDMS after static or dynamic culture for up to 7 days. Quantitative analysis of SEM images for the bacteria attached on SOCAL and S-PDMS have revealed similar results compared to fluorescence images.

### Anti-biofilm Tests against *P. aeruginosa*

**Figure 5:**
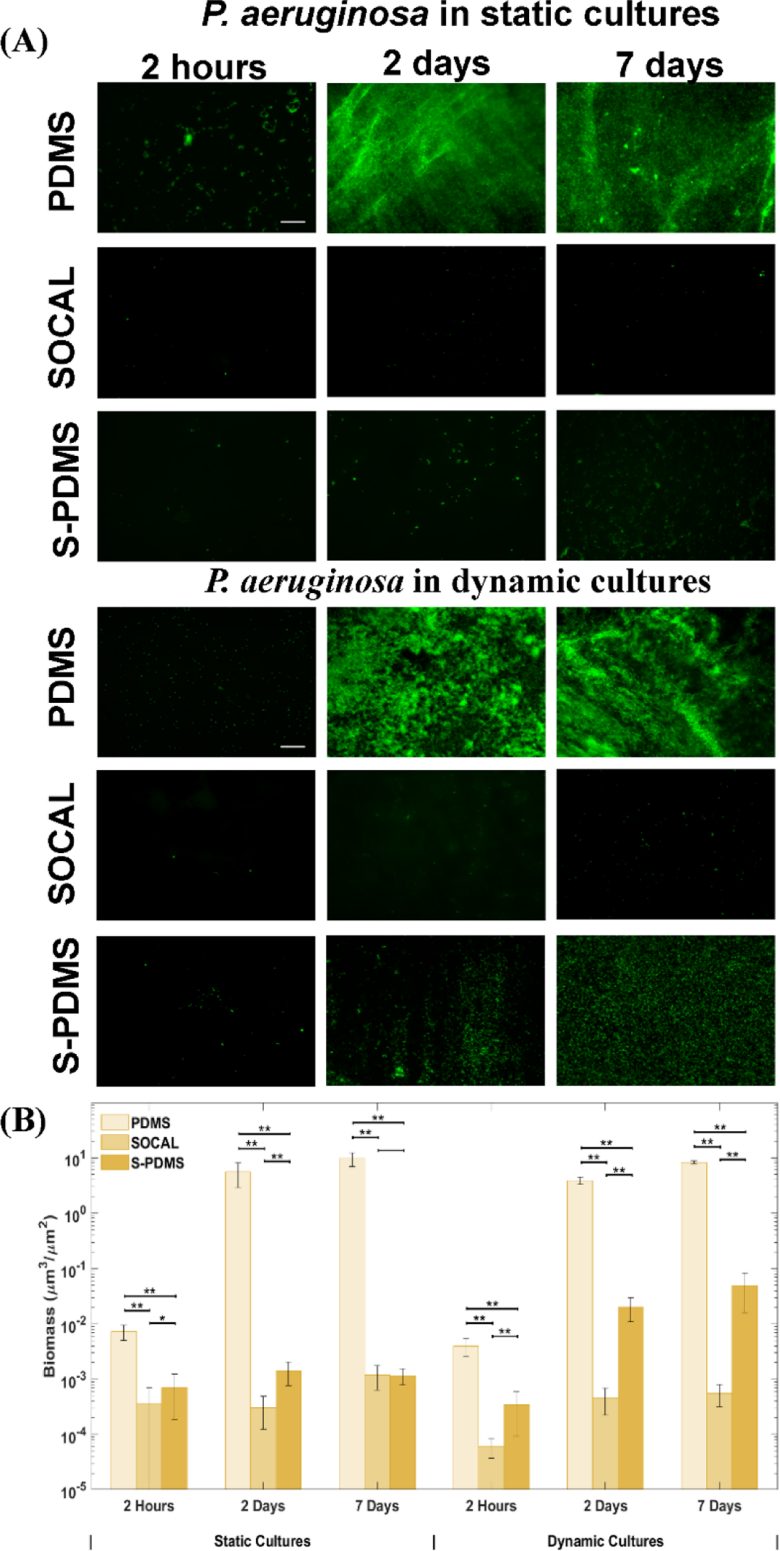
(A) Representative fluorescent images and (B) biomass of the growth of *P. aeruginosa* PAO1 on PDMS, SOCAL, and S-PDMS for 2 h, 2 days, and 7 days in static cell culture and dynamic cell culture. Scale bar = 50 μm for all images. In all cases, 15 images were analyzed for each surface from three independent experiments. Values presented are mean ± SD. *p < 0.05 and **p < 0.001.

*P. aeruginosa* PAO1, a well-characterized strain originally isolated from a wound, was grown on each of the surfaces under static and flow conditions. *P. aeruginosa* initially grew rapidly on the control PDMS surfaces in static culture (Figure 5A). The *P. aeruginosa* biomass appeared mucoid when the PDMS samples from the Petri dish were removed. However, over a longer term (up to 7 days), only sparse and isolated bacteria were found on either SOCAL or S-PDMS (Figure 5A). As seen in Figure 5B, SOCAL and S-PDMS significantly reduced initial bacterial attachment, by 95.0 ± 4.3% or 88.7 ± 11.0%, respectively, compared to the PDMS control. After 2 days, compared to the control surface, the total biomass reduction on the SOCAL and S-PDMS surfaces was over four orders and three orders of magnitude, respectively. Even after 7 days, the total biomass reduction on both SOCAL and S-PDMS surfaces was almost four orders of magnitude less, compared to the control surface (see Figure 5B). However, even though there were significant differences (*p* < 0.05) at 2 h and 2 days, these slippery surfaces performed equally well (*p* = 0.86) in retarding biofilms compared to the PDMS control.

Under flow, *P. aeruginosa* grew significantly over time on the control PDMS surfaces, and dense biofilms were observed after 7 days (Figure 5A). In contrast, throughout the experiment, only sparse and isolated bacteria were found on the SOCAL and S-PDMS surfaces. After 2 h of attachment, SOCAL and S-PDMS led to two orders and one order of magnitude reduction of bacterial adhesion compared to PDMS, respectively (Figure 5B). After the 2 day culture, SOCAL and S-PDMS led to at least three order of magnitude biofilm reduction compared to PDMS. After 7 days of culture in flow, when compared to PDMS, SOCAL and S-PDMS led to greater than four orders of magnitude and two orders of magnitude biofilm reduction, respectively. Throughout the entire dynamic culture period of *P. aeruginosa*, SOCAL significantly outperformed S-PDMS (*p* = 1.1 × 10^–6^ for 2 days and *p* = 5.7 × 10^–5^ for 7 days, respectively).

For *P. aeruginosa*colonization within the initial 2 h, there was a significant difference between static and flow conditions for each surface. After 2 days, there was a significant difference between static and flow conditions for PDMS or S-PDMS but without a significant difference for SOCAL (*p* = 0.06). After 7 days of colonization, there was no significant difference between static and flow conditions for PDMS (*p* = 0.06) but significant differences for either SOCAL (*p* < 0.001) or S-PDMS (*p* < 0.001).

**Figure 6:**
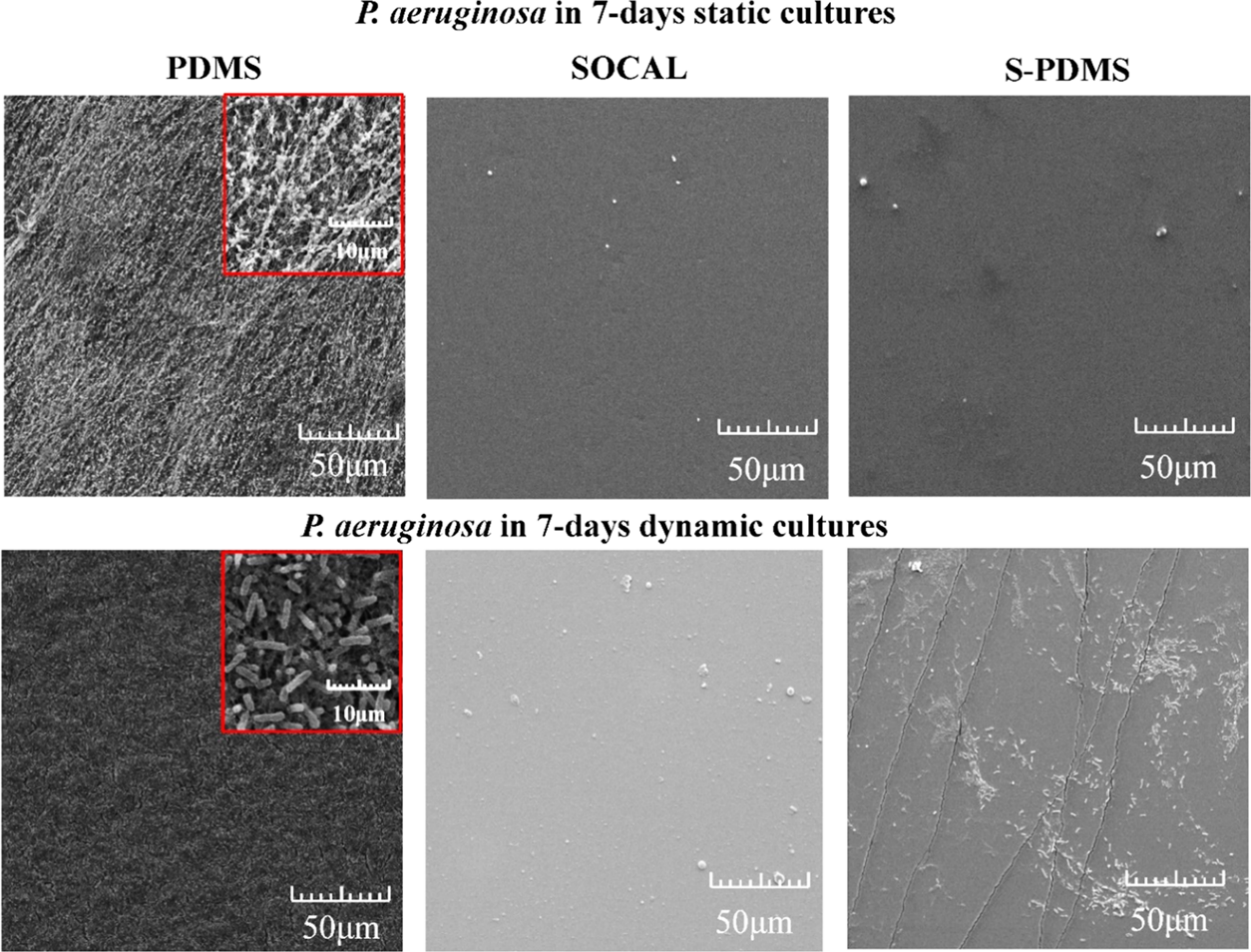
Representative SEM images of 7 day growth of *P. aeruginosa* PAO1 on PDMS, SOCAL, and S-PDMS in static and dynamic cultures. Although dense biofilms were found on PDMS, very few bacteria were found on SOCAL in both static and dynamic cultures. However, more bacteria were found on S-PDMS after 7 days of dynamic culture.

The SEM images (Figure 6) also confirmed that very dense *P. aeruginosa* biofilm growth was apparent on PDMS. Loose fibrous EPS and dense EPS of *P. aeruginosa* biofilms were observed on PDMS for 7 day static and dynamic cultures (see high-resolution images in Figure S2), respectively. By contrast, only sparse bacteria were found on SOCAL or S-PDMS for the 7 day static cultures. After 7 days under dynamic cultures, SOCAL retained excellent antibiofilm characteristics. However, the initial antibiofilm performance for S-PDMS diminished after 7 days, and more bacteria were found compared to SOCAL. Quantitative analysis of SEM images for the bacteria attached on SOCAL and S-PDMS have revealed similar results compared to fluorescence images.

### Biofilm Detachment Tests by Flow

**Figure 7:**
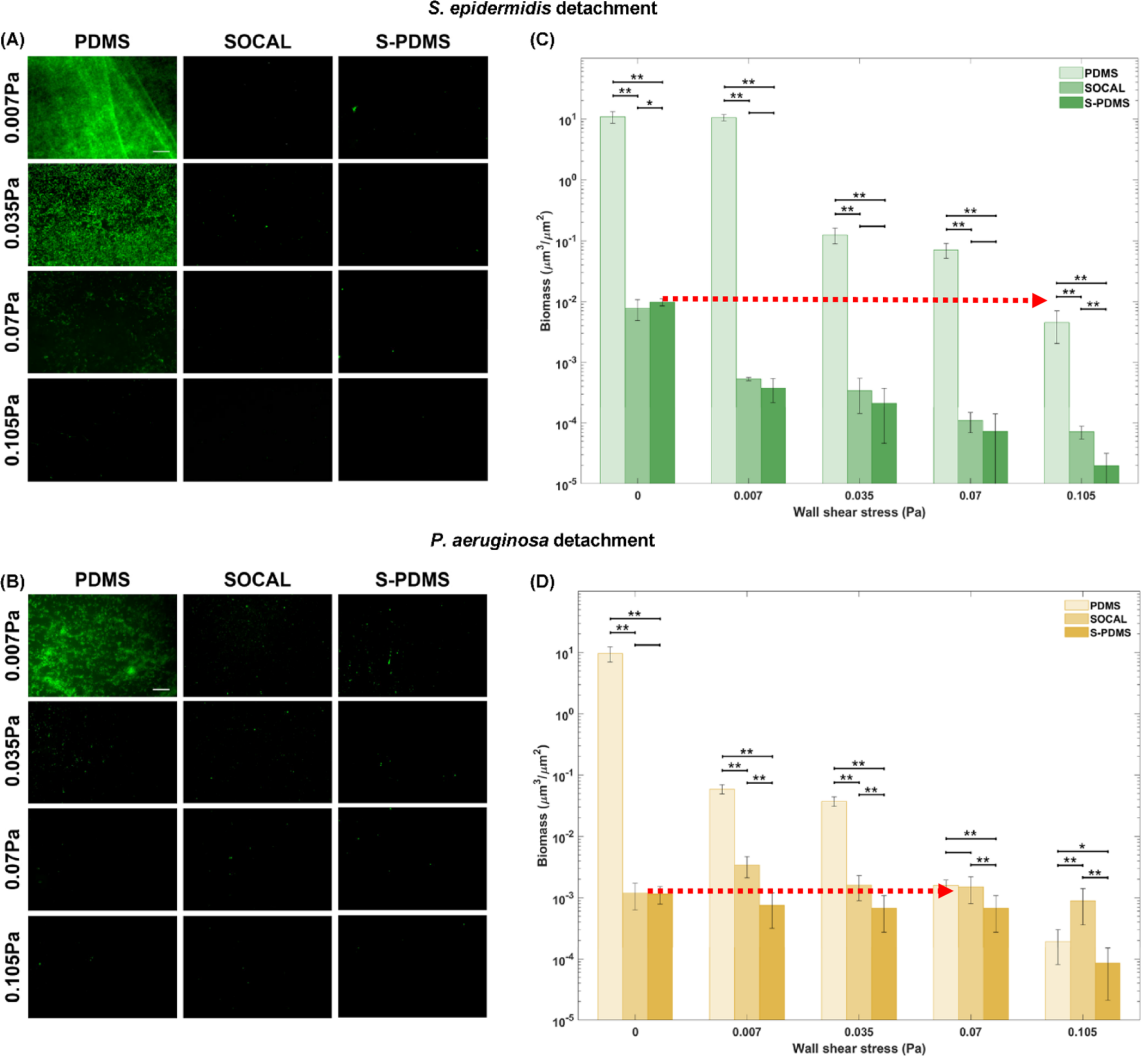
Representative fluorescent images and biomass change with wall shear stress for 7 day biofilms grown in static culture: (A,C) *S. epidermidis* FH8 and (B,D) *P. aeruginosa* PAO1 on PDMS, S-PDMS, and SOCAL after flow shear at 0.007, 0.035, 0.07, and 0.105 Pa. At least six images were analyzed for each surface at each wall shear stress based on three replicates. *p < 0.05 and **p < 0.001.

The detachment results for the pre-grown 7 day biofilms in static culture in Figure 7 also confirmed that even at low shear stress (τ_w_ = 0.007 Pa), 55–68% of *S. epidermidis* bacteria detached from SOCAL and S-PDMS surfaces; however, there was no significant change (*p* = 0.40) in *S. epidermidis* biomass on PDMS. Increasing τ_w_ to 0.07 Pa (almost 10 times the shear stress commonly found in urinary catheters), biomass of *S. epidermidis* on PDMS was still one order of magnitude higher than that of the initial biomass on SOCAL or S-PDMS without flow (Figure 7C). Even at the highest τ_w_ tested (0.1 Pa), the biomass volume of *S. epidermidis* biofilms on PDMS was still several times higher than what was on SOCAL or S-PDMS without flow.

*P. aeruginosa* could be more easily detached from PDMS by applying flow compared to *S. epidermidis*. Increasing τ_w_ to 0.035 Pa (almost five times the shear stress commonly found in urinary catheters), biomass of *P. aeruginosa* on PDMS was still two orders of magnitude higher than that of the initial biomass on SOCAL or S-PDMS without flow (Figure 7D). When τ_w_ reached 0.07 Pa, the biomass volume of *P. aeruginosa* biofilms on PDMS was decreased to that on SOCAL or S-PDMS without flow.

### Reusability Tests after Removing Pre-grown Biomass

After gently wiping off the pre-grown 2 day biomass from SOCAL or S-PDMS, the samples were reused for bacterial growth tests in static cell culture for 7 days. Both surfaces were shown to be reusable without significant difference after wiping off 2 day biofilms (*p* = 0.58 and *p* = 0.29) for *S. epidermidis* from SOCAL and S-PDMS; *p* = 0.92 and *p* = 0.43 for *P. aeruginosa* on SOCAL and S-PDMS, which suggested that both surfaces retained excellent anti-biofilm properties against both *S. epidermidis* and *P. aeruginosa* (see Figure S3 in Supporting Information).

## Discussion

Surface wetting is considered important for bacterial control.^50,51^ For hydrophobic surfaces (CA>90°), very low CAH (CAH < 5°) often indicates strong resistance to bacterial attachment.^27,37^ The SOCAL surfaces fabricated here have low CAH (∼2° on average), which is better than S-PDMS (∼3.2° on average). SOCAL has highly mobile PDMS chains, behaving like a liquid, which are responsible for the very low CAH^41^ and antibacterial adhesion. AFM results have also revealed that SOCAL is over two orders of magnitude softer than PDMS (1:10). It is almost one order of magnitude softer than the solid PDMS with the lowest cross-linker ratio (1:50) ever reported.^52^ If the cross-linker ratio is below 1:50, PDMS can hardly be cross-linked and just flows like liquid. This might also imply that SOCAL is likely to be a liquid-like solid.

Under all conditions tested for 7 days, the SOCAL or S-PDMS surfaces resulted in over two to four orders of magnitude less biofilm formation than PDMS. It is highly unlikely that this inhibition of biofilm formation was due to a bactericidal effect because all three surfaces (PDMS, SOCAL, and S-PDMS) were based on PDMS, which is biocompatible.^15^ This suggests that there was limited bacterial accumulation on SOCAL or S-PDMS or the bacteria were easily detached from the surface.

The antibiofilm results of SOCAL and S-PDMS surfaces presented here were similar to those of other SLIPs reported in the seminal paper by Epstein et al.^28^ In their paper, SLIPS prevented 99.6% of *P. aeruginosa* biofilm formation over a 7 d period under both static and flow conditions. Other studies have also demonstrated that SLIP surfaces are capable of preventing biofilm formation by one–three order of magnitudes for 1–7 day static cultures.^6,37,53,54^ The antibiofilm results of both slippery surfaces in the present study agree well with those of commercial antimicrobial agent-coated materials used for catheters. For example, silver-coated silicone (PDMS) has been shown to reduce *P. aeruginosa* biofilm formation by ∼97% when grown statically for 1 day, compared to pure silicone.^55^ For silicone coated with antibiotics (e.g., rifampin/minocycline, vancomycin, or amikacin), particularly rifampin/minocycline, no significant bacterial colonization was found on these surfaces after 7 days of static culture.^56^ Therefore, the slippery surfaces presented here are possible alternatives, which will not cause antimicrobial resistance but may achieve similar antibiofilm performance.

Furthermore, the wall shear stress required to largely detach pre-grown biomass in static cultures from PDMS, to reach the level of the original biomass on SOCAL and PDMS before flow detachment tests, was above 0.1 Pa and around 0.07 Pa for *S. epidermidis* and *P. aeruginosa*, respectively. These stresses were at least one order of magnitude higher than those found in medical devices (e.g., catheters).^57^

**Figure 8:**
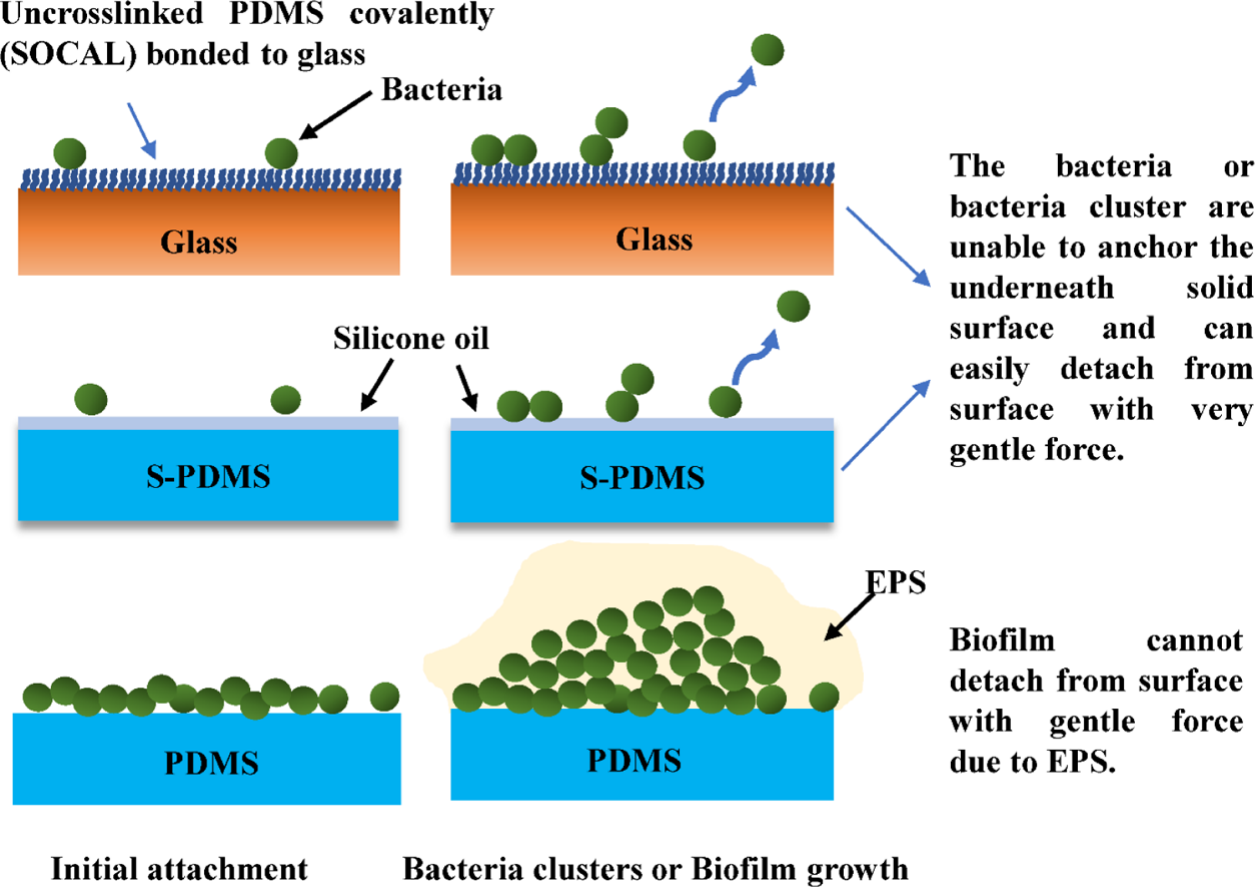
Schematic diagram of bacteria attachment on SOCAL (uncross-linked PDMS covalently bonded to the glass substrate), S-PDMS, and PDMS.

Therefore, we propose the following antibiofilm mechanisms for liquid and liquid-like surfaces (as presented in Figure 8): (1) The ultra-low CAH inhibits initial bacterial attachment. (2) The attached bacteria exhibit a planktonic state when they contact with a liquid or liquid-like surface (i.e., dominated by proliferation with no or little EPS production, as seen in our SEM images). (3) Bacterial cells are unable to establish stable, strong interactions with liquid or liquid-like surfaces, resulting in detachment from the surface during growth or by the action of very gentle external forces. This mechanism would explain why we did not observe cell clusters or biofilms on SOCAL and S-PDMS even after 2 days and 7 days of culture under static and dynamic conditions.

When the oil atop S-PDMS is sufficiently thick, the S-PDMS can have equally good antibiofilm performance similar to SOCAL under all conditions (fresh samples and reused samples after removing 2 day pre-grown biofilms from static cultures). When S-PDMS experienced significant oil loss in flow, it still has similar antibiofilm performance to SOCAL against *S. epidermidis*. However, the antibiofilm performance of S-PDMS against *P. aeruginosa*, after oil depletion in continuous flow for 2–7 days, has decreased significantly by almost two orders of magnitude (*p* < 0.001) compared to SOCAL.

**Figure 9:**
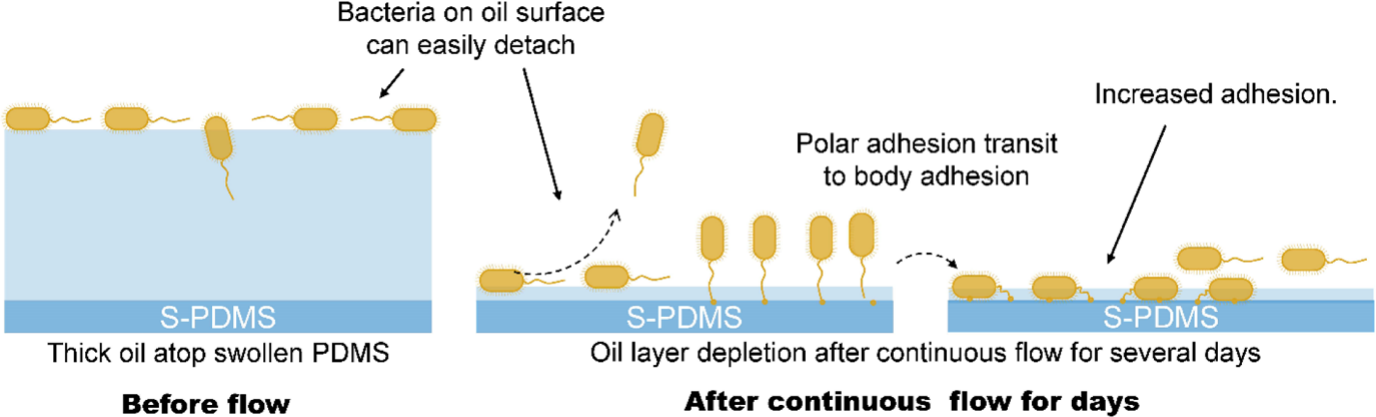
Schematic of *P. aeruginosa* PAO1 attachment on S-PDMS before and after flow-induced oil depletion.

One possibility could be that flow during dynamic culture emphasizes differences in bacterial shape and adhesion appendages such as flagella, which allow polar adhesion^58,59^ and which are present in *P. aeruginosa* but not *S. epidermidis*. The polar adhesion can transit to body adhesion,^58,59^ which may enable stronger attachment (see Figure 9). This is likely to happen for S-PDMS after 7 day flow as the measured oil thickness atop the PDMS surface is less than the cell size of *P. aeruginosa*, which would explain the significantly increased biofilm growth on S-PDMS after 7 days of dynamic culture.

In summary, the liquid-like solid-surface strategy of SOCAL is promising for applications where continuous flow is important, such as catheters. The transparency of visible light is an advantage of this material, which adds value for potential use in other medical devices.

## Materials and Methods

### SOCAL, PDMS, and S-PDMS Fabrication

SOCAL surfaces were created on 25 × 75 mm glass slides using the method detailed by Wang and McCarthy.^41^ The protocol was optimized as described by Armstrong et al.^43^ The clean glass slides were placed in a Henniker plasma cleaner (HPT-100) at 30% power for 20 min, which adds OH bonds to the surface. The slides were then dipped into a reactive solution of isopropanol, dimethyldimethoxysilane, and sulfuric acid (90, 9 and 1% wt) for 5 s and then slowly withdrawn. The slides were then placed in a bespoke humidity chamber in a controlled environment at 60% relative humidity and 25 °C for 20 min. The acid-catalyzed graft polycondensation of dimethyldimethoxysilane creates a homogeneous layer of PDMS chains, grafted onto the surface. The excessive unreacted material was then rinsed away with deionized water, isopropanol, and toluene.

PDMS was used as a control surface because its surface chemistry is similar to SOCAL. To further examine the excellent anti-biofilm performance of SOCAL, tests on swollen PDMS were also performed for comparison. SLIPs were created using S-PDMS. This has a large reservoir of oil compared to LIPs and has demonstrated excellent antibiofilm performance in static culture in recent studies.^37^ S-PDMS has an almost identical chemistry to PDMS and SOCAL, so any impact from surface chemistry will be similar between each of the surfaces.

To prepare PDMS, a mixture of PDMS solution was prepared using a SYLGARD 184 elastomer kit (Dow Corning Corporation, Midland, MI) with a curing agent-to-base ratio of 1:10 (wt/wt). The solution was thoroughly mixed and degassed in a vacuum chamber for 30 min to eliminate air bubbles. The PDMS (∼2 mm thick) was cured in a 37 °C incubator for 1 day. Finally, the cured PDMS sheet was gently cut into 4 cm × 3 cm samples. To prepare S-PDMS, the cured PDMS surfaces were completely immersed in a silicone oil (10 cSt, 0.93 g/mL, Sigma-Aldrich) bath and left for 24 h to allow the oil to fully infiltrate into the PDMS polymer networks. The excess oil was gently removed from the surface by wiping with filter paper. This was carried out to reduce the effects of excess lubricant layers (i.e., wetting ridge^37^) on the following tests.

### Characterization of Slippery Surfaces

The thickness of the oil layer atop the surface of LIPs is typically measured using confocal microscopy, ellipsometry, or by calculation using the weight gained after layering in oil. In our case, however, the refractive index values of silicone oil and PDMS are almost the same, which makes it difficult to quantify the oil thickness of S-PDMS optically. Furthermore, as the oil diffuses into the PDMS, measuring the weight of the swelling oil cannot be used to find the thickness of the surface oil layer. By assuming that the PDMS samples swell isotropically, however, measurements of weight and volume before swelling, after swelling, and after vigorous wiping could be used to approximate the layer thickness and volume of infused oil. By solving a cubic polynomial function, the oil thickness can be calculated. The details have been described in the Supporting Information. Using this approach, we also quantified the oil thickness change after continuous flow for 2 and 7 days.

An in-house goniometer^37,60,61^ was set up to measure the static water CA and CAH under ambient conditions. Advancing angles on slippery surfaces were measured via a syringe pump system (needle gauge size ∼25, water droplet ∼8 μL, with a maximum volume change of 4 μL using the protocol described in ref (48)), and receding angles were measured by withdrawing liquid. CAH was determined as the difference between advancing and receding CAs. At least five measurements were taken.

SOCAL was claimed to be liquid-like coating, which may be expected to be softer than solid PDMS with the lowest cross-linking density. Therefore, nanoindentation tests were performed with AFM using a Flex Bio-AFM system (NanoSurf, Switzerland). A pyramidal AFM cantilever (ContAI-G, BudgetSensors) with a spring constant of *k* ∼ 0.2 N/m was used. The substrate effect is inevitable for nanoindentation of such a thin coating like SOCAL (several nm); therefore, a simple empirical model was used to estimate its modulus (see details in Figure S4 in Supporting Information).

### Flow Cell Setup

Most submerged biofilm formations occur under various flow conditions (e.g., catheters and implant surfaces). Therefore, cell culture was also performed under flow conditions. A parallel-plate flow chamber (PPFC) was designed, where the inlet is sufficiently long to allow fully developed flow, which is important for dynamic culture of bacteria.^62^ A flow cell (length = 10 mm, width = 10 mm, and height = 0.1 mm) made of PDMS was made by pattern molding off a milled acrylic block. This was connected to a syringe pump. The samples (PDMS, S-PDMS, or SOCAL), which were used as a bottom surface, were connected to the top chamber using a press-fit device. In addition, three holes in the flow cell chamber were created: one for pumping broth inoculated with bacteria, another one for fresh tryptic soy broth (TSB) medium, and the third one for collecting waste liquid (see Figure S5 in Supporting Information). Bacterial culture was pumped into the flow chamber until the trapped air had been eliminated, after which the pump was operated for the desired periods of time at 37 °C. When laminar flow is well established in the PPFC, the wall shear rate σ is given using the following equation^63^
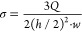
1

The wall shear stress τ_w_ is given using the following formula
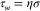
2where *Q* is the volumetric flow rate, *h* and *w* are the height and width of the parallel plate chamber, respectively, and η is the viscosity of the culture medium at 37 °C. TSB culture medium has shown almost the same rheological characteristics to deionised water at 37 °C. Therefore, an average viscosity value of 0.7 mPa·s for TSB culture medium measured using a rheometer (Malvern Kinexus Pro+) was used for the calculation of wall shear stress.

### Bacterial Culture and Antibiofilm Tests

*S. epidermidis* FH8 which was isolated from a chronic rhinosinusitis patient at the Freeman Hospital (Newcastle Upon Tyne) was used.^64^*P. aeruginosa* PAO1, a biofilm-forming bacterial pathogen responsible for many infections,^65^ was also selected. For bacterial adhesion and biofilm formation assays, cells were routinely cultured in TSB (Melford Laboratories Ltd, UK), in a shaker at 180 rpm and 37 °C for 16 h and then diluted to OD600 = 0.2 for *S. epidermidis* FH8 with a spectrophotometer (Biochrom Libra S11, Biochrom Ltd., Cambridge, UK). *P. aeruginosa* PAO1 colonizes on surfaces rapidly. Therefore, to avoid overloading the system, a lower bacterial inoculum (OD600 = 0.01) was chosen for *P. aeruginosa*. Prior to seeding, samples were added to a Petri dish. 20 mL of the diluted bacterial culture was incubated with the PDMS (as control), S-PDMS, and SOCAL surfaces in Petri dish plates (diameter = 10 cm) at 37 °C, for 2 h (bacterial adhesion assay), 2 days, and 7 days (biofilm assay) respectively. For the biofilms developed up to 7 days, half of the TSB medium was changed every 3 days. At the least three independent experiments were performed for each surface type.

Flow is an important factor in many applications and should be considered in assessing antibiofilm effects. For dynamic culture, the syringe pump and the flow cell were placed in a 37 °C incubator. For the 2 h bacterial culture, diluted bacterial-inoculated media (with the same OD in static cell culture) was pumped into the flow cell chamber at a flow rate of 0.01 mL/min (with a Reynolds number of 0.024) and wall shear stress (τ_*w*_) of 0.007 Pa, comparable to typical wall shear stresses in urinary catheters^49^ and ventricular catheters.^57^ For 2 day and 7 day bacterial culture, after 2 h of flow of diluted bacterial-inoculated media, fresh TSB medium was continuously pumped into the flow chamber at the same flow rate (i.e., 0.01 mL/min) at 37 °C.

### Biofilm Detachment Tests

To examine if the bacteria may be weakly attached to the SOCAL and S-PDMS grown under static conditions, biofilm detachment tests were performed in the same parallel-flow cell chambers used for dynamic culture. The 7 day biofilms grown on different surfaces in static culture for 7 days were placed in the parallel-flow chamber, and different flow rates (0.01, 0.05, 0.1, and 0.15 mL/min) were applied for a duration of 1 min. The samples were then removed from the flow chamber for subsequent imaging using a fluorescent microscope (Olympus, BX-61). According to eqs 1 and 2, the resulting wall shear stress (τ_*w*_) at 37 °C ranged from 0.007 to 0.105 Pa, which corresponds to similar wall shear stress in catheters (a few mPa)^57^ and was extended to over an order of magnitude higher to observe trends of biofilm detachment.

### Reuse the Samples after Removing Pre-grown Biofilms

In practice, it will be useful to reuse the antibiofilm surfaces (e.g., non-disposable medical devices or ship hulls) after removing biofilms. To examine the reusability of SOCAL and S-PDMS, the CA and CAH were measured for each surface after removing 7 day pre-grown biofilms formed in static or dynamic culture. The antibiofilm performance of SOCAL and S-PDMS was also tested after wiping off these pre-grown biofilms.

### Biofilm Imaging

Following bacterial adhesion or biofilm formation assays, surfaces were gently rinsed three times with phosphate buffered saline (PBS, pH = 7.4) to remove loosely adhered bacteria. Bacterial cells were stained with Syto9, and fluorescent images were taken on an Olympus BX61 upright fluorescent microscope with a 20× objective lens (N.A. = 0.75). The bacterial cells after 2 h of incubation were visualized by acquiring 2D fluorescent images in a single focal plane. The surface coverage of the bacteria was analyzed using ImageJ [ImageJ (nih.gov)]. Based on the bacteria size for *S. epidermidis* and *P. aeruginosa*, the surface coverage was converted to volume (in COMSTAT software termed biomass) to enable the direct comparisons with longer-period bacteria culture. For biofilms or multi-layered bacteria, z-stacks were taken through the thickness of biofilm from five random locations on the surfaces. The biomass under each field of view was determined using the COMSTAT2 plugin (Lyngby, Denmark) in ImageJ.

To provide insights into possible EPS in biofilms, high-resolution SEM (TESCAN Vega LMU) was used to visualize 7 day biofilm samples grown in both static and dynamic cultures for PDMS, SOCAL, and S-PDMS. The beam voltage and current were set to 8 kV and 62 μA, respectively. Prior to SEM imaging, the samples were washed with PBS and fixed in 2% glutaraldehyde in 3 M Sorenson’s phosphate buffer overnight at 4 °C, which were then transferred to a new plate and dehydrated through a series of ethanol solutions of 25% (v/v), 50%, 75%, and 100%, followed by critical point drying. After critical point drying, the samples were sputter-coated with 5 nm gold coating using a Polaron SEM coating unit.

### Statistical Analysis

Data have been represented as mean values and standard deviations. Student’s *t*-test, assuming unequal variations, was applied, and *p* < 0.05 was considered statistically significant in this study.

## Supporting Information Available

Measurement of oil thickness of S-PDMS, XPS spectrum of SOCAL, high-resolution SEM images of EPS in 7 day *P. aeruginosa* biofilms on PDMS in static and dynamic cultures, comparison of 7 day biofilms grown in static culture for *S. epidermidis* FH8 or *P. aeruginosa* PAO1 on fresh SOCAL or S-PDMS and their re-used counterparts after wiping off pre-grown 2 day biofilms, AFM nanoindentation data analysis of SOCAL, experimental setup for the dynamic culture adopted in this study (PDF)

## Notes

All the data that support the findings of this study are present in the paper and the Supporting Information. Additional data related to this paper may be requested from the authors.

## Abbreviations

EPS: extracellular polymeric substances
CAUTI: catheter-associated urinary tract infections
CRBSIs: catheter-related bloodstream infections
ICUs: intensive care units
SLIPS: slippery liquid-infused porous surface
PDMS: polydimethylsiloxane
S-PDMS: PDMS matrix infused with silicone oil
SOCAL: slippery omniphobic covalently attached liquid-like
CA: contact angle
CAH: contact angle hysteresis

## References

[ref1] Wolcott R. D.; Rhoads D. D.; Bennett M. E.; Wolcott B. M.; Gogokhia L.; Costerton J. W.; Dowd S. E. Chronic Wounds and the Medical Biofilm Paradigm. J. Wound Care 2010, 19, 45–53. 10.12968/jowc.2010.19.2.46966. 20216488

[ref2] Sousa C.; Henriques M.; Oliveira R. Mini-review: Antimicrobial Central Venous Catheters–Recent Advances and Strategies. Biofouling 2011, 27, 609–620. 10.1080/08927014.2011.593261. 21718230

[ref3] Ramstedt M.; Ribeiro I. A. C.; Bujdakova H.; Mergulhão F. J. M.; Jordao L.; Thomsen P.; Alm M.; Burmølle M.; Vladkova T.; Can F.; Reches M.; Riool M.; Barros A.; Reis R. L.; Meaurio E.; Kikhney J.; Moter A.; Zaat S. A. J.; Sjollema J. Evaluating Efficacy of Antimicrobial and Antifouling Materials for Urinary Tract Medical Devices: Challenges and Recommendations. Macromol. Biosci. 2019, 19, 1800384 10.1002/mabi.201800384. 30884146

[ref4] Rupp M. E.; Karnatak R. Intravascular Catheter-Related Bloodstream Infections. Infect. Dis. Clin. 2018, 32, 765–787. 10.1016/j.idc.2018.06.002. 30241718

[ref5] Hollenbeak C. S. The Cost of Catheter-Related Bloodstream Infections: Implications for the Value of Prevention. J. Infus. Nurs. 2011, 34, 309–313. 10.1097/nan.0b013e3182285e43. 21915004

[ref6] Li J.; Kleintschek T.; Rieder A.; Cheng Y.; Baumbach T.; Obst U.; Schwartz T.; Levkin P. A. Hydrophobic Liquid-Infused Porous Polymer Surfaces for Antibacterial Applications. ACS Appl. Mater. Interfaces 2013, 5, 6704–6711. 10.1021/am401532z. 23777668

[ref7] Cao Y.; Su B.; Chinnaraj S.; Jana S.; Bowen L.; Charlton S.; Duan P.; Jakubovics N. S.; Chen J. Nanostructured Titanium Surfaces Exhibit Recalcitrance Towards Staphylococcus epidermidis Biofilm Formation. Sci. Rep. 2018, 8, 1071 10.1038/s41598-018-19484-x. 29348582 PMC5773551

[ref8] Diu T.; Faruqui N.; Sjöström T.; Lamarre B.; Jenkinson H. F.; Su B.; Ryadnov M. G. Cicada-Inspired Cell-Instructive Nanopatterned Arrays. Sci. Rep. 2014, 4, 7122 10.1038/srep07122. 25409910 PMC4238011

[ref9] Bhadra C. M.; Truong V. K.; Pham V. T.; Al Kobaisi M.; Seniutinas G.; Wang J. Y.; Juodkazis S.; Crawford R. J.; Ivanova E. P. Antibacterial Titanium Nano-Patterned Arrays Inspired by Dragonfly Wings. Sci. Rep. 2015, 5, 16817 10.1038/srep16817. 26576662 PMC4649496

[ref10] Fadeeva E.; Truong V. K.; Stiesch M.; Chichkov B. N.; Crawford R. J.; Wang J.; Ivanova E. P. Bacterial Retention on Superhydrophobic Titanium Surfaces Fabricated by Femtosecond Laser Ablation. Langmuir 2011, 27, 3012–3019. 10.1021/la104607g. 21288031

[ref11] Ivanova E. P.; Hasan J.; Webb H. K.; Truong V. K.; Watson G. S.; Watson J. A.; Baulin V. A.; Pogodin S.; Wang J. Y.; Tobin M. J.; Löbbe C.; Crawford R. J. Natural Bactericidal Surfaces: Mechanical Rupture of Pseudomonas aeruginosa Cells by Cicada Wings. Small 2012, 8, 2489–2494. 10.1002/smll.201200528. 22674670

[ref12] Cheng G.; Zhang Z.; Chen S.; Bryers J. D.; Jiang S. Inhibition of Bacterial Adhesion and Biofilm Formation on Zwitterionic Surfaces. Biomaterials 2007, 28, 4192–4199. 10.1016/j.biomaterials.2007.05.041. 17604099 PMC5463736

[ref13] Cheng G.; Li G.; Xue H.; Chen S.; Bryers J. D.; Jiang S. Zwitterionic Carboxybetaine Polymer Surfaces and their Resistance to Long-term Biofilm Formation. Biomaterials 2009, 30, 5234–5240. 10.1016/j.biomaterials.2009.05.058. 19573908 PMC2825140

[ref14] Fu Y.; Jiang J.; Zhang Q.; Zhan X.; Chen F. Robust Liquid-Repellent Coatings based on Polymer Nanoparticles with Excellent Self-cleaning and Antibacterial Performances. J. Mater. Chem. A 2017, 5, 275–284. 10.1039/c6ta06481g.

[ref15] Howell C.; Grinthal A.; Sunny S.; Aizenberg M.; Aizenberg J. Designing Liquid-Infused Surfaces for Medical Applications: A Review. Adv. Mater. 2018, 30, e1802724 10.1002/adma.201802724. 30151909

[ref16] Truong V. K.; Webb H. K.; Fadeeva E.; Chichkov B. N.; Wu A. H. F.; Lamb R.; Wang J. Y.; Crawford R. J.; Ivanova E. P. Air-directed Attachment of Coccoid Bacteria to the Surface of Superhydrophobic Lotus-like Titanium. Biofouling 2012, 28, 539–550. 10.1080/08927014.2012.694426. 22686938

[ref17] Ma J.; Sun Y.; Gleichauf K.; Lou J.; Li Q. Nanostructure on Taro Leaves resists Fouling by Colloids and Bacteria under Submerged Conditions. Langmuir 2011, 27, 10035–10040. 10.1021/la2010024. 21736298

[ref18] Tang P.; Zhang W.; Wang Y.; Zhang B.; Wang H.; Lin C.; Zhang L. Effect of Superhydrophobic Surface of Titanium on Staphylococcus aureus Adhesion. J. Nanomater. 2011, 2011, 1 10.1155/2011/178921.21808638

[ref19] Friedlander R. S.; Vlamakis H.; Kim P.; Khan M.; Kolter R.; Aizenberg J. Bacterial Flagella Explore Microscale Hummocks and Hollows to Increase Adhesion. Proc. Natl. Acad. Sci. 2013, 110, 5624–5629. 10.1073/pnas.1219662110. 23509269 PMC3619351

[ref20] Cao Y.; Jana S.; Bowen L.; Tan X.; Liu H.; Rostami N.; Brown J.; Jakubovics N. S.; Chen J. Hierarchical Rose-Petal Surfaces Delay the Early-stage Bacterial Biofilm Growth. Langmuir 2019, 35, 14670–14680. 10.1021/acs.langmuir.9b02367. 31630525

[ref21] Banerjee I.; Pangule R. C.; Kane R. S. Antifouling Coatings: Recent Developments in the Design of Surfaces That Prevent Fouling by Proteins, Bacteria, and Marine Organisms. Adv. Mater. 2011, 23, 690–718. 10.1002/adma.201001215. 20886559

[ref22] Chen X.; Wen G.; Guo Z. What are the Design Principles, from the Choice of Lubricants and Structures to the Preparation Method, for a Stable Slippery Lubricant-Infused Porous Surface?. Mater. Horiz. 2020, 7, 1697–1726. 10.1039/d0mh00088d.

[ref23] Lafuma A.; Quéré D. Slippery Pre-Suffused Surfaces. Europhys. Lett. 2011, 96, 56001 10.1209/0295-5075/96/56001.

[ref24] Wong T.-S.; Kang S. H.; Tang S. K. Y.; Smythe E. J.; Hatton B. D.; Grinthal A.; Aizenberg J. Bioinspired Self-Repairing Slippery Surfaces with Pressure-Stable Omniphobicity. Nature 2011, 477, 443–447. 10.1038/nature10447. 21938066

[ref25] Jamil M. I.; Ali A.; Haq F.; Zhang Q.; Zhan X.; Chen F. Icephobic Strategies and Materials with Superwettability: Design Principles and Mechanism. Langmuir 2018, 34, 15425–15444. 10.1021/acs.langmuir.8b03276. 30445813

[ref26] Wei C.; Zhang G.; Zhang Q.; Zhan X.; Chen F. Silicone Oil-Infused Slippery Surfaces based on Sol–Gel Process-Induced Nanocomposite Coatings: A Facile Approach to Highly Stable Bioinspired Surface for Biofouling Resistance. ACS Appl. Mater. Interfaces 2016, 8, 34810–34819. 10.1021/acsami.6b09879. 27998125

[ref27] Sotiri I.; Tajik A.; Lai Y.; Zhang C. T.; Kovalenko Y.; Nemr C. R.; Ledoux H.; Alvarenga J.; Johnson E.; Patanwala H. S.; Timonen J. V. I.; Hu Y.; Aizenberg J.; Howell C. Tunability of Liquid-Infused Silicone Materials for Biointerfaces. Biointerphases 2018, 13, 06D401 10.1116/1.5039514. 30092645

[ref28] Epstein A. K.; Wong T.-S.; Belisle R. A.; Boggs E. M.; Aizenberg J. Liquid-Infused Structured Surfaces with Exceptional Anti-Biofouling Performance. Proc. Natl. Acad. Sci. 2012, 109, 13182–13187. 10.1073/pnas.1201973109. 22847405 PMC3421179

[ref29] Zhao L.; Li R.; Xu R.; Si D.; Shang Y.; Ye H.; Zhang Y.; Ye H.; Xin Q. Antifouling slippery liquid-infused membrane for separation of water-in-oil emulsions. J. Membr. Sci. 2020, 611, 118289 10.1016/j.memsci.2020.118289.

[ref30] Ouyang Y.; Zhao J.; Qiu R.; Hu S.; Chen M.; Wang P. Liquid-Infused Superhydrophobic Dendritic Silver Matrix: A Bio-Inspired Strategy to Prohibit Biofouling on Titanium. Surf. Coat. Technol. 2019, 367, 148–155. 10.1016/j.surfcoat.2019.03.067.

[ref31] Xiao Y.; Zhao J.; Qiu R.; Shi Z.; Niu S.; Wang P. Slippery Liquid-Infused Surface from Three-Dimensional Interconnecting Net Structure Via Breath Figure Approach and its Usage for Biofouling Inhibition. Prog. Org. Coat. 2018, 123, 47–52. 10.1016/j.porgcoat.2018.06.012.

[ref32] Kratochvil M. J.; Welsh M. A.; Manna U.; Ortiz B. J.; Blackwell H. E.; Lynn D. M. Slippery Liquid-Infused Porous Surfaces that Prevent Bacterial Surface Fouling and Inhibit Virulence Phenotypes in Surrounding Planktonic Cells. ACS Infect. Dis. 2016, 2, 509–517. 10.1021/acsinfecdis.6b00065. 27626103 PMC5198836

[ref33] Tenjimbayashi M.; Park J. Y.; Muto J.; Kobayashi Y.; Yoshikawa R.; Monnai Y.; Shiratori S. In Situ Formation of Slippery-Liquid-Infused Nanofibrous Surface for a Transparent Antifouling Endoscope Lens. ACS Biomater. Sci. Eng. 2018, 4, 1871–1879. 10.1021/acsbiomaterials.8b00134. 33445342

[ref34] Ware C. S.; Smith-Palmer T.; Peppou-Chapman S.; Scarratt L. R. J.; Humphries E. M.; Balzer D.; Neto C. Marine Antifouling Behavior of Lubricant-Infused Nanowrinkled Polymeric Surfaces. ACS Appl. Mater. Interfaces 2018, 10, 4173–4182. 10.1021/acsami.7b14736. 29250952

[ref35] Yong J.; Chen F.; Yang Q.; Fang Y.; Huo J.; Zhang J.; Hou X. Nepenthes Inspired Design of Self-Repairing Omniphobic Slippery Liquid Infused Porous Surface (SLIPS) by Femtosecond Laser Direct Writing. Adv. Mater. Interfac. 2017, 4, 1700552 10.1002/admi.201700552.

[ref36] Zhou X.; Lee Y.-Y.; Chong K. S. L.; He C. Superhydrophobic and Slippery Liquid-Infused Porous Surfaces Formed by the Self-Assembly of a Hybrid ABC Triblock Copolymer and their Antifouling Performance. J. Mater. Chem. B 2018, 6, 440–448. 10.1039/c7tb02457f. 32254523

[ref37] Cao Y.; Jana S.; Tan X.; Bowen L.; Zhu Y.; Dawson J.; Han R.; Exton J.; Liu H.; McHale G.; Jakubovics N. S.; Chen J. Antiwetting and Antifouling Performances of Different Lubricant-Infused Slippery Surfaces. Langmuir 2020, 36, 13396–13407. 10.1021/acs.langmuir.0c00411. 33141589

[ref38] Peppou-Chapman S.; Neto C. Mapping Depletion of Lubricant Films on Antibiofouling Wrinkled Slippery Surfaces. ACS Appl. Mater. Interfaces 2018, 10, 33669–33677. 10.1021/acsami.8b11768. 30168715

[ref39] Adera S.; Alvarenga J.; Shneidman A. V.; Zhang C. T.; Davitt A.; Aizenberg J. Depletion of Lubricant from Nanostructured Oil-Infused Surfaces by Pendant Condensate Droplets. ACS Nano 2020, 14, 8024–8035. 10.1021/acsnano.9b10184. 32490664

[ref40] Howell C.; Vu T. L.; Johnson C. P.; Hou X.; Ahanotu O.; Alvarenga J.; Leslie D. C.; Uzun O.; Waterhouse A.; Kim P.; Super M.; Aizenberg M.; Ingber D. E.; Aizenberg J. Stability of Surface-Immobilized Lubricant Interfaces under Flow. Chem. Mater. 2015, 27, 1792–1800. 10.1021/cm504652g.

[ref41] Wang L.; McCarthy T. J. Covalently Attached Liquids: Instant Omniphobic Surfaces with Unprecedented Repellency. Angew. Chem., Int. Ed. Engl. 2016, 55, 244–248. 10.1002/anie.201509385. 26568536

[ref42] Krumpfer J. W.; Bian P.; Zheng P.; Gao L.; McCarthy T. J. Contact Angle Hysteresis on Superhydrophobic Surfaces: An Ionic Liquid Probe Fluid Offers Mechanistic Insight. Langmuir 2011, 27, 2166–2169. 10.1021/la105068c. 21271691

[ref43] Armstrong S.; McHale G.; Ledesma-Aguilar R.; Wells G. G. Pinning-Free Evaporation of Sessile Droplets of Water from Solid Surfaces. Langmuir 2019, 35, 2989–2996. 10.1021/acs.langmuir.8b03849. 30702296

[ref44] Louette P.; Bodino F.; Pireaux J.-J. Poly(dimethyl siloxane) (PDMS) XPS Reference Core Level and Energy Loss Spectra. Surf. Sci. Spectra 2005, 12, 38–43. 10.1116/11.20050908.

[ref45] Chen J.; Bull S. J. On the Factors Affecting the Critical Indenter Penetration for Measurement of Coating Hardness. Vacuum 2009, 83, 911–920. 10.1016/j.vacuum.2008.11.007.

[ref46] McHale G.; Orme B. V.; Wells G. G.; Ledesma-Aguilar R. Apparent Contact Angles on Lubricant-Impregnated Surfaces/SLIPS: From Superhydrophobicity to Electrowetting. Langmuir 2019, 35, 4197–4204. 10.1021/acs.langmuir.8b04136. 30759342

[ref47] Semprebon C.; McHale G.; Kusumaatmaja H. Apparent Contact Angle and Contact Angle Hysteresis on Liquid Infused Surfaces. Soft Matter 2017, 13, 101–110. 10.1039/c6sm00920d. 27221773

[ref48] Barrio-Zhang H.; Ruiz-Gutiérrez É.; Armstrong S.; McHale G.; Wells G. G.; Ledesma-Aguilar R. Contact-Angle Hysteresis and Contact-Line Friction on Slippery Liquid-like Surfaces. Langmuir 2020, 36, 15094–15101. 10.1021/acs.langmuir.0c02668. 33258609 PMC8016194

[ref49] Nowatzki P. J.; Koepsel R. R.; Stoodley P.; Min K.; Harper A.; Murata H.; Donfack J.; Hortelano E. R.; Ehrlich G. D.; Russell A. J. Salicylic Acid-Releasing Polyurethane Acrylate Polymers as Anti-Biofilm Urological Catheter Coatings. Acta Biomater. 2012, 8, 1869–1880. 10.1016/j.actbio.2012.01.032. 22342353

[ref50] Werb M.; Garcia C. F.; Bach N. C.; Grumbein S.; Sieber S. A.; Opitz M.; Lieleg O. Surface Topology Affects Wetting Behavior of Bacillus Subtilis Biofilms. npj Biofilms Microbiomes 2017, 3, 11 10.1038/s41522-017-0018-1. 28649412 PMC5460217

[ref51] Jahed Z.; Shahsavan H.; Verma M. S.; Rogowski J. L.; Seo B. B.; Zhao B.; Tsui T. Y.; Gu F. X.; Mofrad M. R. K. Bacterial Networks on Hydrophobic Micropillars. ACS Nano 2017, 11, 675–683. 10.1021/acsnano.6b06985. 28045495

[ref52] Brown X. Q.; Ookawa K.; Wong J. Y. Evaluation of Polydimethylsiloxane Scaffolds with Physiologically-Relevant Elastic Moduli: Interplay of Substrate Mechanics and Surface Chemistry Effects on Vascular Smooth Muscle Cell Response. Biomaterials 2005, 26, 3123–3129. 10.1016/j.biomaterials.2004.08.009. 15603807

[ref53] MacCallum N.; Howell C.; Kim P.; Sun D.; Friedlander R.; Ranisau J.; Ahanotu O.; Lin J. J.; Vena A.; Hatton B.; Wong T.-S.; Aizenberg J. Liquid-infused Silicone as a Biofouling-Free Medical Material. ACS Biomater. Sci. Eng. 2014, 1, 43–51. 10.1021/ab5000578. 33435082

[ref54] Kovalenko Y.; Sotiri I.; Timonen J. V. I.; Overton J. C.; Holmes G.; Aizenberg J.; Howell C. Bacterial Interactions with Immobilized Liquid Layers. Adv. Healthcare Mater. 2017, 6, 1600948 10.1002/adhm.201600948. 27930872

[ref55] Wang R.; Neoh K. G.; Kang E. T.; Tambyah P. A.; Chiong E. Antifouling Coating with Controllable and Sustained Silver Release for Long-Term Inhibition of Infection and Encrustation in Urinary Catheters. J. Biomed. 2015, 103, 519–528. 10.1002/jbm.b.33230. 24922113

[ref56] Li H.; Fairfax M.; Dubocq F.; Darouiche R. O.; Rajpurkar A.; Thompson M.; Tefilli M. V.; Dhabuwala C. B. Antibacterial Activity of Antibiotic Coated Silicone Grafts. J. Urol. 1998, 160, 1910–1913. 10.1097/00005392-199811000-00082. 9783984

[ref57] Lee S.; Kwok N.; Holsapple J.; Heldt T.; Bourouiba L. Enhanced Wall Shear Stress Prevents Obstruction by Astrocytes in Ventricular Catheters. J. R. Soc. Interface 2020, 17, 20190884 10.1098/rsif.2019.0884. 32603649 PMC7423414

[ref58] Berne C.; Ellison C. K.; Ducret A.; Brun Y. V. Bacterial Adhesion at the Single-Cell Level. Nat. Rev. Microbiol. 2018, 16, 616–627. 10.1038/s41579-018-0057-5. 30008468

[ref59] Li G.; Brown P. J. B.; Tang J. X.; Xu J.; Quardokus E. M.; Fuqua C.; Brun Y. V. Surface Contact Stimulates the Just-In-Time Deployment of Bacterial Adhesins. Mol. Microbiol. 2012, 83, 41–51. 10.1111/j.1365-2958.2011.07909.x. 22053824 PMC3245333

[ref60] Gart S.; Mates J. E.; Megaridis C. M.; Jung S. Droplet Impacting a Cantilever: A Leaf-Raindrop System. Phys. Rev. Appl. 2015, 3, 044019 10.1103/physrevapplied.3.044019.

[ref61] Huhtamäki T.; Tian X.; Korhonen J. T.; Ras R. H. Surface-Wetting Characterization Using Contact-Angle Measurements. Nat. Protoc. 2018, 13, 1538 10.1038/s41596-018-0003-z. 29988109

[ref62] Bakker D. P.; van der Plaats A.; Verkerke G. J.; Busscher H. J.; van der Mei H. C. Comparison of Velocity Profiles for Different Flow Chamber Designs Used in Studies of Microbial Adhesion to Surfaces. Appl. Environ. Microbiol. 2003, 69, 6280–6287. 10.1128/aem.69.10.6280-6287.2003. 14532092 PMC201240

[ref63] Elimelech M. Particle Deposition on Ideal Collectors from Dilute Flowing Suspensions- Mathematical Formulation, Numerical-Solution, and Simulations. Sep. Technol. 1994, 4, 186–212. 10.1016/0956-9618(94)80024-3.

[ref64] Shields R. C.; Mokhtar N.; Ford M.; Hall M. J.; Burgess J. G.; ElBadawey M. R.; Jakubovics N. S. Efficacy of a Marine Bacterial Nuclease Against Biofilm Forming Microorganisms Isolated from Chronic Rhinosinusitis. PLoS One 2013, 8, e55339 10.1371/journal.pone.0055339. 23441151 PMC3575374

[ref65] Cole S. J.; Lee V. T. Cyclic Di-GMP Signaling Contributes to Pseudomonas aeruginosa-Mediated Catheter-Associated Urinary Tract Infection. J. Bacteriol. 2016, 198, 91–97. 10.1128/jb.00410-15. 26195591 PMC4686188

