# Supplementary material for: Slippery Liquid-Like Solid Surfaces with Promising Antibiofilm Performance under Both Static and Flow Conditions
Source: ACS Appl Mater Interfaces. 2022 Jan 31;14(5):6307–19. doi: 10.1021/acsami.1c14533 (PMC9096797; doi:10.1021/acsami.1c14533)
Supplement: Supplementary file 1 — am1c14533_si_001.pdf [file am1c14533_si_001.pdf]

Supporting Information

Slippery Liquid-Like Solid Surfaces with Promising Antibiofilm Performance in both Static and Flow Conditions

Yufeng Zhu[1], Glen McHale[2], Jack Dawson[1], Steven Armstrong[2], Gary Wells[2], Rui Han[1], Hongzhong Liu[3], Waldemar Vollmer[4], Paul Stoodley[5,6], Nicholas Jakubovics[7], Jinju Chen[1].*

[1] School of Engineering, Newcastle University, Newcastle Upon Tyne, NE1 7RU, UK. [2] School of Engineering, University of Edinburgh, Edinburgh, EH9 3FB, UK. [3] School of Mechanical Engineering, Xi'an Jiaotong University, Xi'an 710054, China; [4] Centre for Bacterial Cell Biology, Biosciences Institute, Newcastle University, Newcastle upon Tyne, NE2 4AX, UK. [5] Department of Microbial Infection and Immunity and the Department of Orthopaedics, The Ohio State University, Columbus, OH, 43210, USA. [6] National Centre for Advanced Tribology at Southampton (nCATS), National Biofilm Innovation Centre (NBIC), Mechanical Engineering, University of Southampton, Southampton, S017 1BJ, UK. [7] School of Dental Sciences, Faculty of Medical Sciences, Newcastle University, Newcastle Upon Tyne, NE2 4BW, UK.

*Corresponding author. Email: Jinju.chen@ncl.ac.uk

Measurement of oil thickness for S-PDMS

The weight (w1) of S-PDMS after a gentle wipe (the same protocol to prepare S-PDMS for surface wetting measurements and antibiofilm tests) was measured. The weight (w2) and the dimensions (x,y,z) of the swollen PDMS (after vigorously wiping off all surface oil from S-PDMS) was then measured. A total of 10 replicates were used for the measurements.

Due to the nature of physical absorption, the oil thickness (t) is assumed uniform in all directions. The oil thickness is then determined using following equation[1]

$$\frac{w_1 - w_2}{oildensity} = (x+2t)(y+2t)(z+2t) - xyz \qquad \text{(S1)}$$

where x,y,z are the length, width and height of the swollen PDMS substrate, respectively. The oil density used in this study was $0.93 g/cm^3$. A matlab code was written to determine oil thickness for various S-PDMS before flow, after 2-days and 7-days flow.

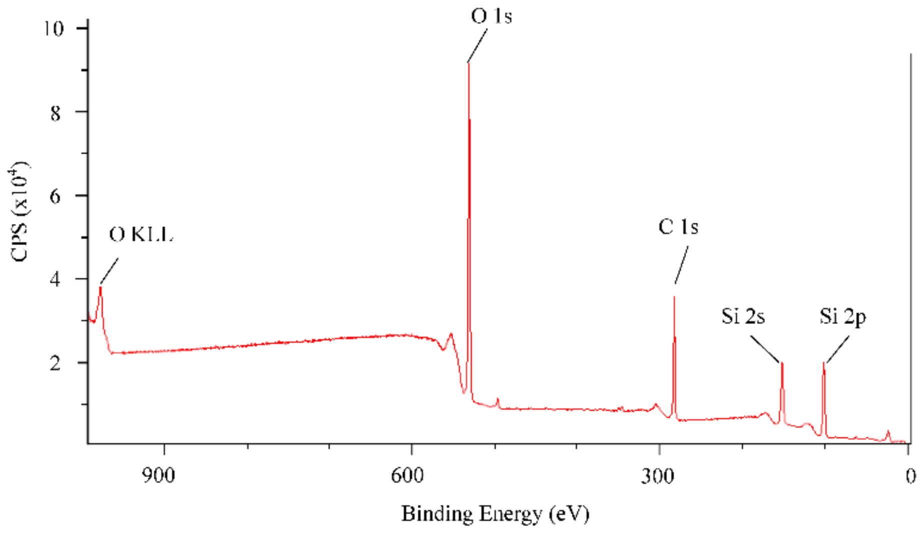

Figure S1.

The XPS spectrum of SOCAL. It shows the similar spectra to PDMS[2].

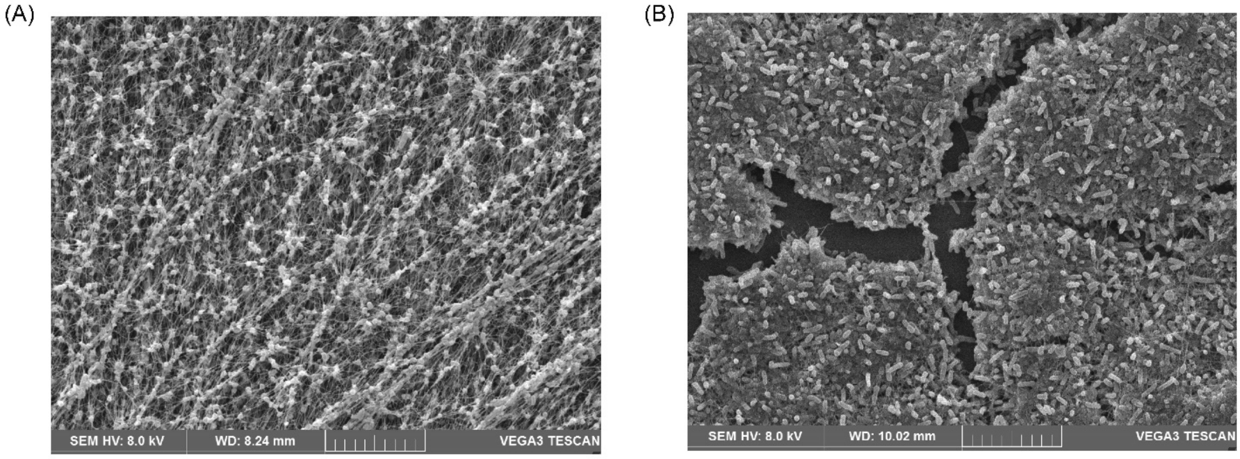

Figure S2.

High resolution SEM images of *P. aeruginosa* PAO1 after 7-days (A) static and (B) dynamic cultures. The scale bar is 10μm. Loose and dense EPS were found, respectively.

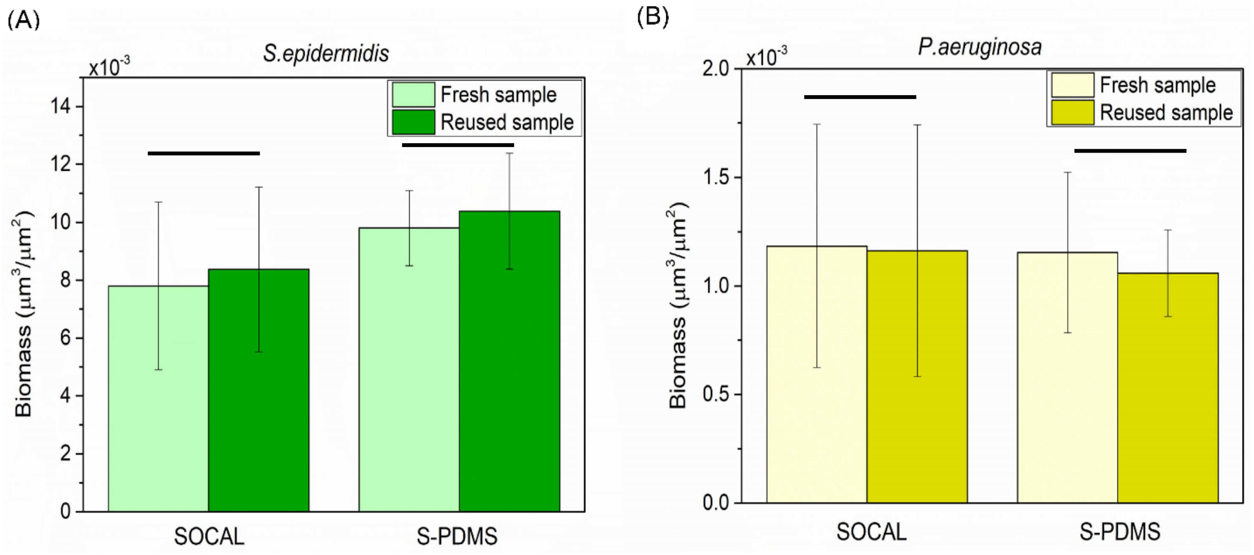

Figure S3.
Comparison of 7-day biofilms grown in static culture: (A) *S. epidermidis* FH8 and (B) *P. aeruginosa* PAO1 on fresh SOCAL or S-PDMS and their re-used counterparts after wiping off pre-grown 2-days biofilms. There is no significant difference for biomass between fresh samples and their re-used counterparts for both SOCAL and S-PDMS. In all cases, 15 images were analyzed for each surface from 3 independent experiments.

AFM nanoindentation data analysis of SOCAL

SOCAL has exhibited viscoelastic properties which may be approximated by a Prony series model. Therefore, the following indentation model was adopted to analyse the force-displacement curves (*F*- *h*) for nanoindentation tests[3-5].

$$F = \frac{2}{\pi}\frac{E_0}{(1-\upsilon^2)}\tan(\alpha)\, h^2[g_\infty + g_1 \frac{\tau}{t}(1 - \exp{(-\frac{t}{\tau})})] \tag{S2}$$

$$g_\infty + g_1 = 1 \tag{S3}$$

$$E = E_0\, g_\infty \tag{S4}$$

where E is Young's modulus of the samples, α is semi-included angle of an indenter (25° in this study), ν is Poisson's ratio which is approximately 0.5, t is the loading time and τ is time constant.

MATLAB code was written to determine the Young's modulus. At least 30 curves were analyzed for each indentation depth. The apparent modulus-indentation depth curve was fitted to an exponential function, and the extrapolated module for SOCAL is about 8.8kPa (see Fig. S4).

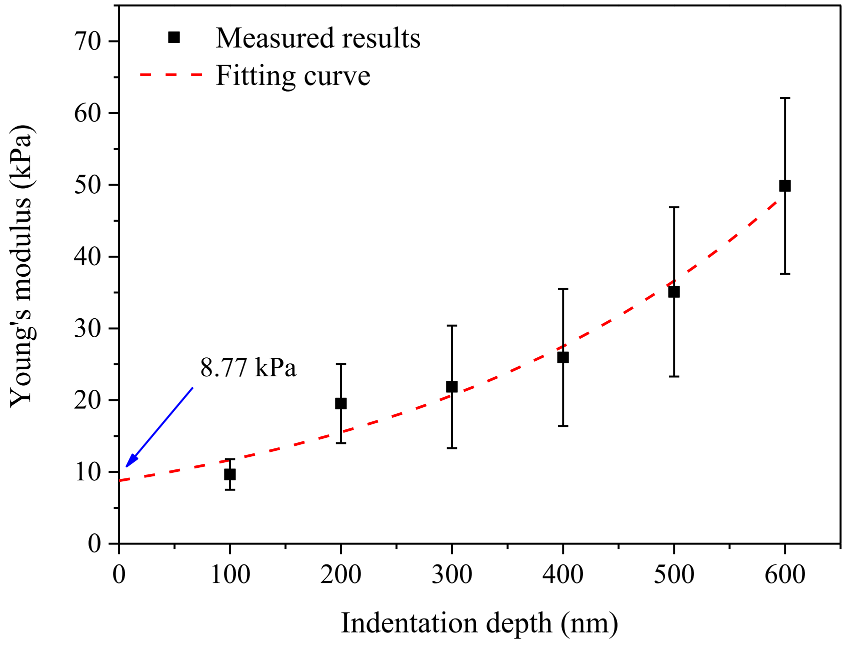

Figure S4. The measured apparent Young's modulus at different indentation depth. The extrapolated modulus was an estimated modulus of SOCAL.

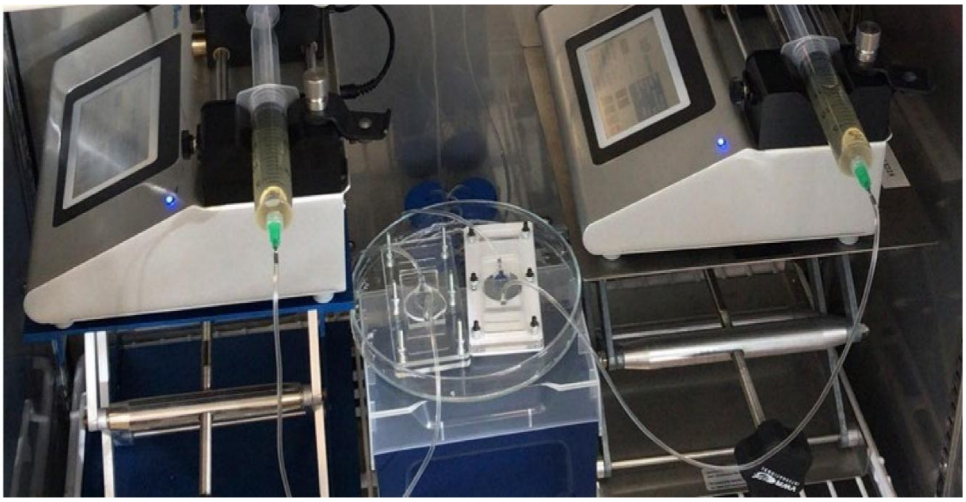

Figure S5.
The experimental set-up for the dynamic culture adopted in this study.

(1) Cao, Y.; Jana, S.; Tan, X.; Bowen, L.; Zhu, Y.; Dawson, J.; Han, R.; Exton, J.; Liu, H.; McHale, G.; Jakubovics, N. S.; Chen, J. Antiwetting and Antifouling Performances of Different Lubricant-Infused Slippery Surfaces. *Langmuir* **2020,** *36* (45), 13396–13407.
(2) Louette, P.; Bodino, F.; Pireaux, J. J. Poly(dimethyl siloxane) (PDMS) XPS Reference Core Level and Energy Loss Spectra. *Surf. Sci. Spectra* **2005,** *12*, 38-43.
(3) Chen, J. J.; Lu, G. X. Finite Element Modelling of Nanoindentation based Methods for Mechanical Properties of Cells. *J. Biomech.* **2012,** *45* (16), 2810-2816.
(4) Chen, J. J. Nanobiomechanics of Living Cells: a Review. *Interface Focus* **2014,** *4* (2), No. 20130055.
(5) Chen, J.; Wright, K. E.; Birch, M. A. Nanoscale Viscoelastic Properties and Adhesion of Polydimethylsiloxane for Tissue Engineering. *Acta Mech Sin.* **2014,** *30* (1), 2-6.
